# Case Report: Intragastric balloon placement for weight loss in LVAD patients—a bridge to heart transplantation

**DOI:** 10.3389/fcvm.2025.1579218

**Published:** 2025-05-07

**Authors:** Aarti Desai, Shriya Sharma, ADJ Siaw, Jose Ruiz, Victoria Gómez, Rohan Goswami

**Affiliations:** ^1^ Division of Heart Failure and Transplant, Mayo Clinic, Jacksonville, FL, United States; ^2^ Department of Quantitative Health Sciences, Mayo Clinic, Jacksonville, FL, United States; ^3^ Department of Gastroenterology and Hepatology, Mayo Clinic, Jacksonville, FL, United States

**Keywords:** LVAD, heartMate 3, Heart transplant, obesity, intragastric balloon, endoscopic bariatric therapies, heart failure

## Abstract

Obesity significantly increases the risk of advanced heart failure, complicating heart transplantation candidacy. Despite aggressive medical therapies, achieving weight loss in these patients remains challenging, especially in patients after durable ventricular assist device (LVAD). More intense weight loss interventions such as bariatric and metabolic surgery and endoscopic bariatric therapies (EBTs) can lead to meaningful weight reduction, enabling previously ineligible individuals to become transplant candidates. A 51-year-old gentleman with end-stage heart and kidney failure status-post HeartMate 3 LVAD (Abbott, Chicago, IL) and Class II obesity (BMI 36.5 kg/m^2^), was deemed ineligible for heart transplant due to high BMI (≥35 kg/m^2^). Despite lifestyle modification, he was unable to lose weight, and BMI increased to 40.8 kg/m^2^ over the next 10 months. A multi-disciplinary discussion was held to discuss possible weight loss options, and after careful consideration, bariatric surgery was not deemed safe. The decision was made to proceed with EBTs, and an intragastric balloon (IGB) was successfully placed as a bridge to heart transplant. The IGB was removed at the six-month period per standard of care, and the patient had lost 16.5 Kg, achieving a 12.4% Total Body Weight Loss with a BMI of 35.3 kg/m^2^. The patient underwent successful heart and kidney transplant and is now two months post-transplant. His BMI 2-months post-transplant is 37 kg/m^2^. This case highlights the feasibility and efficacy of EBT therapy with IGB placement as an alternative to bariatric surgery for patients with LVAD placement and significant comorbidities who need to lose clinically significant weight to be deemed eligible for heart transplant.

## Highlights

• When patients are deemed ineligible for transplantation due to significant obesity (BMI ≥35 Kg/m^2^) and not fit for bariatric surgery, EBTs such as IGB therapy could be considered as safe and effective alternative for weight loss.
• The ability to place IGB endoscopically in patients with LVAD requiring anticoagulation and inotrope therapies enhances its appeal as a weight loss option in this group of previously limited patients.
• We discuss perioperative anticoagulation strategies, anesthesia complications and intraoperative LVAD management in depth.

## 1 Introduction

Obesity is among the most significant risk factors leading to advanced heart failure. With every 1 kg/m^2^ increase in Body Mass Index (BMI), the risk of heart failure (HF) increases by 5% in men and 7% in women (1). Among HF patients that require heart transplantation (HT), obesity is known to increase the post-transplant risk of rejection, infections, kidney dysfunction, graft vasculopathy and all-cause mortality (2). As per the 2024 ISHLT guidelines for the for the Evaluation and Care of Cardiac Transplant Candidates, a BMI of <35 kg/m^2^ is preferred to reduce wait times and increase survival rates, hence, most heart transplant centers have a strict BMI cut-off at ≤35 kg/m^2^ (3, 4).

Candidates for HT often present with severe hemodynamic instability and must achieve rapid weight reduction to meet transplant eligibility criteria. Weight loss is particularly challenging in patients with HF due to impaired exercise tolerance and volume overload. Bariatric surgeries and endoscopic therapies have reported successful weight loss reduction, thereby allowing otherwise ineligible individuals to achieve heart transplant candidacy. Moreover, these procedures have also demonstrated an increase in life expectancy compared to patients managed with only medical weight loss therapies (1). Considering such favorable outcomes, bariatric surgeries have gained momentum among transplant candidates.

Patients with end-stage HF are often bridged with mechanical circulatory support (MCS) devices to support cardiac function. Left ventricular assist devices (LVAD), the most used MCS devices, have become an integral part of HF therapy either as a bridge to HT or as destination therapy. Patients with LVAD pose additional risks to surgical procedures, predominantly due to long-term anticoagulation therapy and hemodynamic shifts caused by the device and anesthesia. Infection, bleeding (most commonly gastrointestinal bleeding), stroke, thrombosis, thromboembolism, and risks of anesthesia are the most prevalent peri-operative complications encountered. Up to 42.5% of patients undergoing surgical procedures while on LVAD support require transfusion due to bleeding and still have a 56.5% mortality rate (5). Endoscopic bariatric therapies (EBTs) are nonsurgical, less invasive weight loss interventions that result in clinically significant weight loss and improvement in comorbidities. Intragastric Balloon (IGB) is a space-occupying device that is endoscopically placed in the gastric body that lead to early satiety and delayed gastric emptying, resulting in lower total caloric intake and weight loss (6). As an endoscopic procedure, it may present lower risk profile than patients undergoing invasive bariatric surgery particularly in the context of anticoagulation and device management in patients with an LVAD (6).

We present the case of a 51-year-old gentleman with advanced HF status post LVAD placement and a BMI of 40.8 kg/m^2^ who was not able to achieve weight reduction with exercise, fluid management, and semaglutide injections (5, 7). He underwent successful IGB therapy with clinically meaningful weight loss as a bridge to transplant.

## 2 Case presentation

A 51-year-old gentleman was transferred to our facility with profound cardiogenic shock (LVEF <15%) due to biventricular failure secondary to nonischemic cardiomyopathy managed previously with multiple MCS devices including Impella CP, Intra-aortic balloon pump (IABP), and Impella 5.5. History is also significant chronic kidney disease stage 4 treated with peritoneal dialysis and continuous renal replacement therapy, hypertension, biventricular implantable cardioverter-defibrillator, diabetes mellitus, and obesity (BMI 36.5 kg/m^2^).

The patient was ineligible for heart transplant candidacy due to high BMI (criteria ≥35 kg/m^2^) and the decision was made to support the patient with HeartMate 3 (Abbott, Chicago, IL) (HM3) LVAD with hopes of optimization of his weight and volume status to achieve future transplant eligibility. The patient's weight loss was challenging despite lifestyle modification with regular exercise, vigilance on volume status, and implementation of anti-obesity pharmacotherapy with semaglutide weekly injections. Over the next 10 months, the patient's BMI continued to rise reaching as high as 40.8 kg/m^2^. Given his cardiac disease and frail status, he was not deemed a suitable candidate for bariatric surgery. A multidisciplinary discussion was held, and the decision was made to proceed with IGB therapy in combination with lifestyle modification as a means to lose significant weight.

At the time of pre-operative evaluation, the LVAD showed adequate function, with a speed 5,700 RPM, flow rate 5.7 L/min, power 4.6 watts, pulsatility index of 3.2, and a mean arterial pressure of 81 mmHg. He was taking warfarin 4 mg daily and aspirin 81 mg daily with an INR 1.8. The patient was admitted prior to the endoscopic intervention with the IGB to undergo IV heparin bridging. Guideline-directed medical therapy (GDMT) was continued with amiodarone 200 mg OD, spironolactone 25 mg OD, empagliflozin 10 mg OD, and hydralazine 50 mg TID with volume status controlled via dialysis.

**Figure 1:**
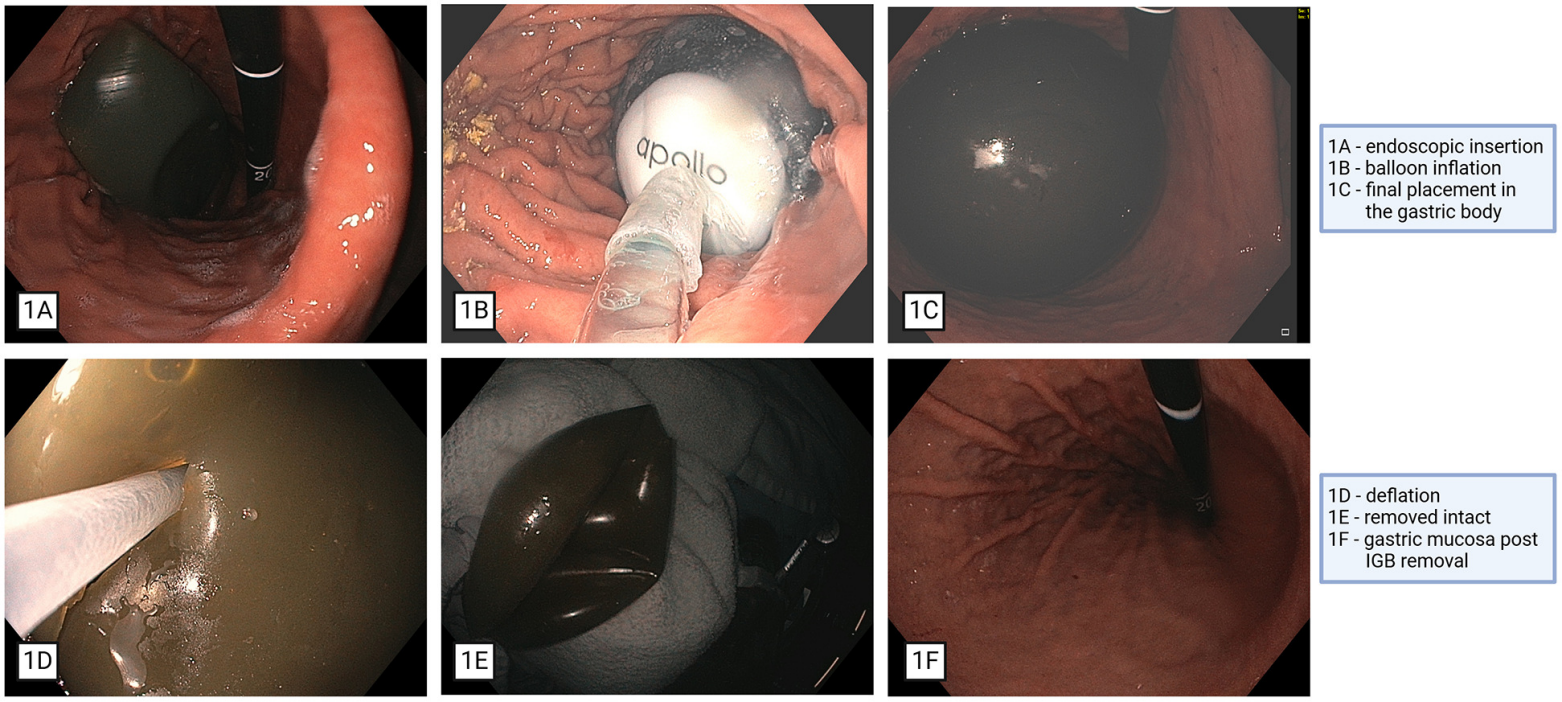
Endoscopic placement and removal of intragastric balloon. IGB, intragastric balloon.

IGB placement was performed under general endotracheal anesthesia (GETA) using a standard adult gastroscope and following standard protocol for placement of the device. Heparin (12 U/kg/hr) infusion was stopped 5 hours prior to the procedure and resumed the following day post IGB placement. After undergoing upper endoscopy, the IGB, preloaded on a catheter in the deflated state, was introduced through the mouth and situated in the gastric body under endoscopic guidance. The IGB (ORBERA, Apollo Endosurgery, TX, USA) was instilled with 550 ml of a solution of saline and methylene blue and was deployed in the stomach (Figures 1A–C). Perioperative measures were implemented to reduce post-procedure nausea and vomiting as well as mucosal injury from the balloon, following an established institution-directed pharmacotherapy protocol. LVAD function and hemodynamics remained stable throughout the procedure. He remained hospitalized for 2 days to reinitiate warfarin 4 m BD and allow his INR to return to the therapeutic range of 2–3 and had no concern for post operative bleeding.

His post-discharge course was complicated by severe nausea and vomiting resulting from a pseudo-obstruction on Day 10 caused by movement of the balloon in the stomach leading to malpositioning within the antrum. It required endoscopic repositioning of the balloon which was uneventful.

**Figure 2:**
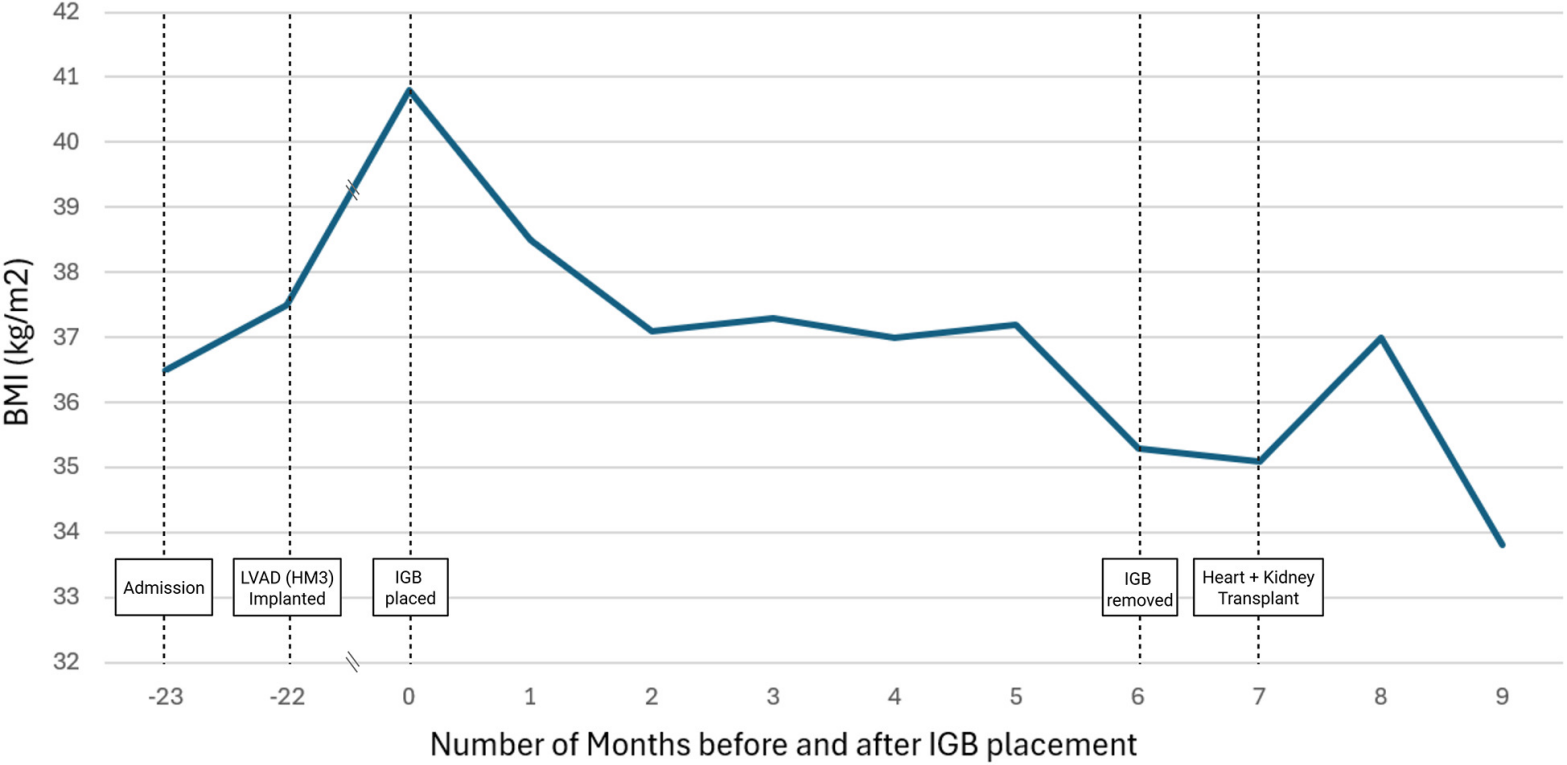
Changes in BMI pre- and post-intragastric balloon placement. BMI, body mass index (kg/m^2^); HM3, HeartMate 3; IGB, intragastric balloon; LVAD, left ventricular assist device.

The patient tolerated IGB therapy without complications and the balloon was subsequently removed at the 6-month period with another upper endoscopy with GETA. The balloon was punctured with a dedicated needle, the fluid contents of the balloon aspirated, and the deflated balloon removed with forceps (Figures 1D–F). At the time of IGB removal the patient's BMI was 35.3 kg/m^2^. The patient lost 16.5 Kg, a 12.4% Total Body Weight Loss (TWL) and BMI reduction of 5.5 kg/m^2^ (Figure 2).

Right heart catheterization performed 1 month post-IGB removal with dobutamine 7.5 mcg/kg/min support revealed elevated biventricular filling pressures [MAP 63 mmHg, RA 16 mmHg, RV 64/10 mmHg, PA 77/29 (mean 45) mmHg, PCWP 35 mmHg, Fick CO 2.9 L/min and Fick CI 1.2 L/min/m^2^] with worsening group 2 pulmonary hypertension (PVR 2.4 WU) and severe aortic regurgitation resulting in poor LV unloading despite LVAD support. The heart team decided his aortic insufficiency was a contraindication for IABP and it would not be safe to have an Impella device placed with concurrent HM3 support.

Given ongoing hemodynamic compromise despite aggressive medical treatments and weight loss efforts while on Dobutamine and HM3 support, a UNOS status 1 exception was submitted, and he was listed for both heart and kidney transplant and successfully received both 30 days later.

The patient is currently on his immunosuppressive regimen with Tacrolimus 9 mg BID (targeted trough 8–10), mycophenolate 500 mg daily, and prednisone 10 mg daily. At 2 months post-transplant, he remains stable with delayed renal graft function and no signs of rejection.

## 3 Discussion

There is robust evidence supporting the association between obesity and HF, however, weight management is an evolving challenge, particularly as new mechanical circulatory devices reshape the management of HF. LVAD serves as a bridge to transplant in many patients while waiting for a suitable donor match. However, LVAD support is associated with an increase in appetite, limited exercise tolerance and reduced metabolism, all leading to weight gain (8). Additionally, patients with obesity supported by LVAD demonstrate an increased risk of right HF and acute kidney injury (8). Achieving weight loss in LVAD patients is essential for hemodynamic optimization and improved outcomes, however, it is particularly challenging in this population due to decreased baseline exercise tolerance and device associated discomfort.

### 3.1 Current weight management options for patients with LVAD

Lifestyle modification with diet and exercise, anti-obesity pharmacotherapy and bariatric surgery have served as the main standard of interventions for patients with LVADs requiring weight loss. Studies focusing on exercise regimen and dietary modifications such as calorie restriction, intermittent fasting and ketogenic diets reveal modest improvements or mixed results (8). This may be attributed to poor exercise tolerance and underlying cardiac metabolic derangement. The key barriers to conservative weight management therapies in HF patients are compliance and sustainability.

Lifestyle modification alone may result in mild to modest at most, weight loss. However, it is not sustainable long-term. The summit trial and other studies using GLP1 analogs have demonstrated a 12%–21% weight loss in HFpEF patients (9, 10). Additionally, GLP1 mediated weight loss is also associated with a lower morbidity and functional improvements in 6MWT and quality of life (9). However, these medications are costly and remain a challenge for insurance coverage.

Bariatric surgery remains as one of the most effective weight loss interventions, showing benefit across all spectrums of patient populations, including those with HF. A metanalysis of bariatric procedures conducted on LVAD patients that included 14 studies and 29 patients, with 83% laparoscopic sleeve gastrectomy and 17% Roux-en-Y gastric bypass showed a BMI reduction from 45.5 ± 6.6 to 31.4 ± 19.3 kg/m^2^. 47% of these patients were successfully transplanted (11). However, these results were not without risks: 39.3% patients had adverse events within 30 days including GI bleeding, infections and staple line leakage (11). Bariatric surgeries in this patient population present heightened risk due to the interplay of factors including risks associated with anesthesia, anticoagulation, and procedural risks. Furthermore, bariatric surgery may not be deemed safe in certain patients with HF, and patients may not desire such invasive weight loss interventions.

In this context, EBTs are nonsurgical, less invasive and effective weight loss interventions that significantly gained momentum and popularity due to their effectiveness and safety profile. EBTs are traditionally categorized as space occupying, gastric remodeling, or small bowel focused interventions. One type of space occupying therapy with an intragastric balloon, entails use of a space occupying device and is performed with a brief endoscopic procedure. IGB therapy has shown excellent weight loss in the general population as well as in patients with HF. A case series of 2 patients reported a 10–14.4% TWL reducing BMI to ≤35 kg/m^2^ over the 6-month therapy period to achieve transplant eligibility and eventually a successful HT. Successful IGB therapy in patients awaiting liver transplant has been reported, however, there are limited case reports and no large-scale studies reporting the use of IGB therapy for weight loss in patients with advanced HF with existing LVAD as a bridge to transplant eligibility (6).

### 3.2 Transplant eligibility

The current UNOS guidelines advise against HT for patients with pre-transplant BMI ≥35 kg/m^2^ due to difficulty finding suitable donors resulting in prolonged waitlist times and increased post-transplant morbidity and mortality. Weight loss is particularly difficult in patients with end-stage HF and existing LVADs due to functional limitations and volume overload despite aggressive diet control. This is exemplified by our patient, who adhered strictly to his diet and exercise regimen and used semaglutide for 6 months without significant weight loss. TWL of 12.4% achieved by our LVAD patient align closely with the average TWL of 12.2% reported for IGB use in the general population (12).

### 3.3 Perioperative anticoagulation strategy

The use of aspirin and warfarin to prevent pump thrombosis in LVAD patients requires careful consideration due to the associated increased risk of intraoperative bleeding (13). While bariatric surgeries such as sleeve gastroplasty/gastrectomy, Roux-en-Y gastric bypass, gastric band, or biliopancreatic diversion yield greater total weight loss (>50% TWL), these invasive procedures pose increased intraoperative risk of bleeding in patients with LVADs taking anticoagulants. Hence, IGBs may be preferred due to lower bleeding risks associated with endoscopic procedures for which anticoagulation is not an absolute contraindication (14). After multidisciplinary consultation, our patient was admitted to discontinue warfarin and undergo heparin bridging 2 days before the procedure. Post-IGB placement, we restarted systemic anticoagulant therapy following a standard protocol. Monitoring with aPTT or anti-Xa was implemented to maintain therapeutic anticoagulation while overlapping with oral vitamin K antagonist (warfarin) until target INR of 2–3 was reached. Continuous infusion of systemic anticoagulation was stopped when INR was above 1.8. Monitoring for bleeding and thrombosis was performed before patient discharge with daily CBC and lactate dehydrogenase.

### 3.4 Intraoperative LVAD management

It is considered safe to administer general anesthesia in LVAD patients provided adequate care is taken to monitor hemodynamic shifts as LVAD function is sensitive to preload and afterload changes (13). Inadequate volume optimization can decrease preload leading to inadequate LV filling, while the anesthetic agent may induce vasodilation, resulting in decreased afterload and increased LV offloading. Both factors can lead to LV collapse and may require speed reduction, fluids, or vasopressin. RV failure may lead to a similar outcome and may be indirectly observed as a decrease in CVP and hypotension despite medical therapies (13). Additionally, intraoperative patient position should be considered to prevent a mechanical kink in the outflow graft, accidental disconnection, or preload changes. A multidisciplinary team of individuals experienced in managing LVAD complications should be present during and after the procedure.

## 4 Conclusions

EBTs such as IGB therapy are nonsurgical and less invasive weight loss interventions that may provide a safe and effective alternative for patients with LVAD therapy who are not qualified or deemed fit for bariatric surgery. Given that this particular patient population requires anticoagulation and inotrope support, the low invasiveness of IGB placement and removal enhances its appeal as a weight loss option in this group of limited patients. Large-scale studies and trials studying outcomes and safety of EBTs in these more frail patient populations are needed to help create algorithms for clinically significant weight management.

## Data Availability

The raw data supporting the conclusions of this article will be made available by the authors, without undue reservation.
